# The Effect of Inferior Rectus Muscle Thickening on Intraocular Pressure in Thyroid-Associated Ophthalmopathy

**DOI:** 10.1155/2021/9736247

**Published:** 2021-01-29

**Authors:** Xiuhong Li, Xu Bai, Zhibin Liu, Ming Cheng, Jia Li, Nian Tan, Hongfeng Yuan

**Affiliations:** Department of Ophthalmology, Daping Hospital, Army Medical University, No. 10,Changjiang Branch,Daping,Yuzhong District, Chongqing, China

## Abstract

**Objective:**

We aimed to evaluate the effect of inferior rectus muscle thickening on intraocular pressure (IOP) in patients with thyroid-associated ophthalmopathy (TAO).

**Materials and Methods:**

We analyzed 33 patients with TAO (50 eyes) who presented with hypotropia in the primary position. There was significant eyeball movement restriction and inferior rectus muscle thickening was confirmed on computed tomography or magnetic resonance imaging. We measured IOP changes in patients with TAO and normal participants in the primary position and upgaze using Icare tonometer. Moreover, we measured the preoperative and postoperative IOP in 13 patients with hypotropia who underwent inferior rectus recession.

**Results:**

The average inferior rectus thickness in the TAO and control groups was 0.71 ± 0.13 mm and 0.36 ± 0.12 mm, respectively. In the TAO group, the mean IOP was 16.8 ± 2.1 mm Hg in primary position (hypotropia), which increased by 8.9 ± 2.7 mm Hg to 25.7 ± 3.1 mm Hg in upgaze (horizontal vision). In the control group, the mean IOP in the primary position (horizontal vision) was 15.1 ± 1.9 mm Hg, which increased by 2.5 ± 1.4 mm Hg to 17.6 ± 2.1 mm Hg in upgaze. Compared with normal participants, patients with TAO who presented inferior rectus muscle thickening had a significantly greater increase in the IOP (*P* < 0.0001). In the patients with TAO who underwent inferior rectus muscle recession, there was a postoperative reduction in the IOP in the horizontal vision by 9.4 ± 5.2 mm Hg.

**Conclusion:**

Inferior rectus muscle thickening in patients with TAO causes a significant increase in the IOP upon upgaze compared to that in normal individuals. Given the ease of misdiagnosis as glaucoma, IOP measurement in patients with TAO should be performed in the primary position.

## 1. Introduction

Thyroid-associated ophthalmopathy (TAO) is the most prevalent orbital disease. Its characteristic clinical features include exophthalmos, eyelid retraction, ocular dyskinesia, exposure keratitis, and ocular hypertension. In 1897, Brailey and Eyre [1] for the first time reported that TAO could cause ocular hypertension. Algvere et al. [2] reported a 5%–24% prevalence of TAO with ocular hypertension. The pathogenesis underlying increased IOP in TAO remains unclear with the following possible mechanisms being proposed. For example, increased intraorbital inflammation, edema, as well as fat and muscle volume could lead to increased episcleral venous pressure, which results in venous outflow obstruction and, in turn, causes increased IOP responsiveness [3, 4]. Other mechanisms include increased mucopolysaccharide deposition in the trabecular meshwork and increased aqueous humor outflow resistance [5], as well as eyeball compression by fibrotic or thickened rectus muscles [6]. Moreover, it could be associated with predisposition to glaucoma [7, 8]. Therefore, many patients with TAO are diagnosed with glaucoma since they present ocular hypertension [9] with a majority being treated with anti-glaucoma drugs for IOP reduction [10, 11]. Moreover, some of the patients undergo anti-glaucoma surgery [11], including trabeculectomy, drainage device implantation, laser surgery, and even CLASS surgery.

In most patients with TAO, the inferior rectus muscle is involved. Inferior rectus muscle thickening causes downward turning of the eyeball, which results in restricted hypotropia. We previously observed that patients with TAO who presented with hypotropia had greater IOP than those without hypotropia. This could be attributed to the need for the eyeball to upgaze in patients with hypotropia to achieve horizontal vision required for IOP measurement using a noncontact tonometer. Few studies have assessed the IOP in patients with TAO who present with hypotropia (the inferior strabismus in primary position), as well as changes in IOP during eye movements in patients with TAO. Therefore, in this study, we analyzed patients with TAO who had simple inferior rectus muscle thickening in the non-inflammatory phase. Specifically, we aimed to assess their IOP in primary position (hypotropia) and upgaze (horizontal vision) and explore the effect of inferior rectus muscle thickening on IOP in patients with TAO.

## 2. Methods and Materials

### 2.1. Clinical Characteristics of the Patients

All patients with TAO were examined in the Department of Ophthalmology, Daping Hospital, Army Medical University, between January 1^st^ 2018, and May 31^st^ 2019. Moreover, we included patients with no orbital disease in the control group and patients who underwent inferior rectus muscle recession in the surgery group. The inclusion criteria were as follows: (1) a diagnosis of TAO; (2) limited upward eye movement; (3) inferior rectus muscle thickening confirmed by computed tomography (CT) or magnetic resonance imaging (MRI); and (4) no glaucoma history. The exclusion criteria were (1) a glaucoma history; (2) all-directional ocular movement limitation; and (3) no significant inferior rectus muscle thickening on orbital MRI or CT.

### 2.2. Materials and Methods

We performed IOP measurements using Icare Pro tonometer (TAO 3, Icare Finland Oy company, Helsinki, Finland). With the patients with TAO in a sitting position, the IOP was measured in primary position (hypotropia) and then at upgaze position. In the control group, the IOP was measured in the primary position (horizontal vision) and then at maximum upgaze eyeball displacement. In the inferior rectus muscle recession group, the IOP was measured at 3 days and 2 weeks before and after the operation, respectively. IOP measurements were performed six consecutive times. After removing the highest and lowest values, the average of the remaining four measurements was obtained. Consequently, the observation indicators were IOP at different positions in the TAO and control groups and preoperative and postoperative IOP in the surgery group.

### 2.3. Statistical Analysis

We recorded and collated the obtained measurement using Excel tables. All statistical analyses were performed using SPSS 23.0 software. The results were presented as mean ± standard deviation. Independent samples were compared using the Mann–Whitney *U*‐test, and statistical significance was set at *P* < 0.05.

## 3. Results

**Figure 1:**
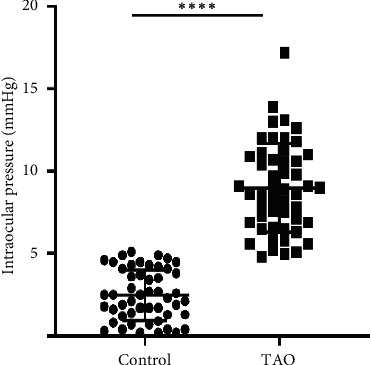
The scatter plot of the distribution of the IOP difference between the upgaze and primary positions in the TAO and control groups.

Table 1 presents the demographic data. The mean IOP in the 36 enrolled patients with TAO (50 eyes) in the primary (hypotropia) and upgaze (horizontal vision) positions were 16.8 ± 2.1 mm Hg and 25.7 ± 3 mm Hg, respectively. In the 33 normal participants (50 eyes), the mean IOP in the primary and upgaze positions were 15.1 ± 1.9 mm Hg and 17.6 ± 2.1 mm Hg, respectively. As shown in Table 2, there was a significant between-group difference in the IOP (*P* < 0.0001).  Figure 1 presents the scatter plot of the distribution of the IOP difference between the upgaze and primary positions in the TAO and control groups.

**Figure 2:**
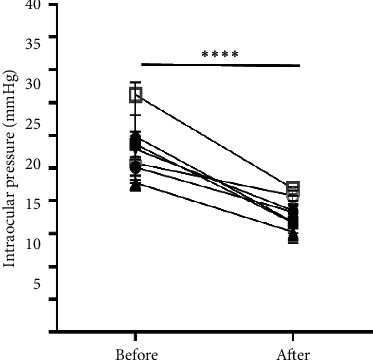
The mean preoperative and postoperative IOP in horizontal vision are 27.4 ± 4.6 mmHg and 18.0 ± 2.7 mm Hg.

Among the 13 patients with TAO who underwent inferior rectus muscle recession for inferior rectus muscle thickening and hypotropia in the primary position, the mean preoperative and postoperative IOP in horizontal vision were 27.4 ± 4.6 mm Hg and 18.0 ± 2.7 mm Hg, respectively (Figure 2). This represented a significant postoperative IOP decrease of 9.4 mm Hg (*P* < 0.0001).

## 4. Discussion

Patients with TAO present different clinical symptoms that vary from the active to the inactive phase. There is a difference between muscle thickening and increased orbital fat, as well as between single muscle thickening and multiple muscle thickening. Therefore, the underlying mechanism underlying increased IOP in patients with TAO remains unclear. Fishman and Benes [12, 13] reported an IOP increase in patients with TAO at the upgaze position. Nardi et al. [14] performed IOP measurements in the primary position and with 22° up-down displacement of the strabismus. The IOP in the upgaze position in normal participants and patients with TAO ranged from 0 to +3 mm Hg and +1 to +15 mm Hg, respectively. Spierer and Eisenstein [15] reported that compared with normal participants, patients with TAO presented with an increased and similar IOP in the active and inactive phase, respectively. Saunders et al. [16] measured the IOP in the primary position and at all-directional maximum eyeball rotations. The IOP in upgaze position in normal individuals and patients with TAO ranged from +3 to +10 mm Hg and >10 mm Hg, respectively. Differences in the previous findings could be attributed to the use of different tonometers, inclusion criteria, and eye position measurements. Therefore, to minimize the confounding effect of several factors, we analyzed patients with TAO with simple inferior rectus muscle thickening in the inactive phase. This was to determine the effect of inferior rectus muscle thickening on IOP in patients with TAO. The inferior rectus muscle is the most affected in patients with TAO [17]. Moreover, these patients are often misdiagnosed with glaucoma. The Icare tonometer used in this study allows quick and accurate IOP measurement in patients with hypotropia. Schreiber et al. [18] reported a good correlation between the Icare and Goldmann tonometers and that the Icare tonometer yielded reliable results.

We found that IOP in patients with TAO who presented with inferior rectus muscle thickening and hypotropia was in the normal range (about 17.5 Mm Hg) at the primary position. However, it significantly increased (by 8.9 mm Hg to about 25.8 mm Hg) at upgaze. In the control group, the IOP in the primary and upgaze positions were within the normal range (<21 mm Hg; nonsignificant increase upon upgaze of about 2.5 mm Hg). Compared with normal controls, patients with TAO presented with a significantly higher IOP increase. The underlying mechanism could involve inferior rectus muscle thickening. Specifically, in the upgaze position, the pulling of the coarsened fibrous inferior rectus muscle in patients with TAO causes the superior rectus muscle to use greater force to upgaze the eyeball. This additional force acts on the eyeball wall and the compression of the superior and inferior rectus muscle significantly increases the IOP [19, 20]. In normal people, the inferior rectus muscle is relaxed when the eyeball is upgazed with little force required from the superior rectus muscle. Therefore, there is a small IOP increase when the eyeball is upgazed in normal individuals.

The IOP in patients with TAO who present hypotropia was not high in the primary position (hypotropia). However, it increased by 8.3 mm Hg upon upgaze, which easily exceeds the 21 mm Hg standard. Currently, most ophthalmological clinics in hospitals use noncontact tonometers for IOP measurement, which requires patients to horizontally look forward. In patients with TAO who present hypotropia, the measured IOP is that of the upward transposition rather than the primary position; therefore, they are often misdiagnosed with glaucoma. However, the IOP in these patients is usually low when the eyeballs are downward positioned. The patients often upgaze when required to look forward or upward rather than rotate their eyes upward since this makes them uncomfortable. These patients do not present glaucoma since the eyeball is in primary position for long periods and there is no optic nerve and retina ischemia. It has been reported that lack of TAO treatment can result in high IOP within 3 years, suspicious glaucoma within 8 years and glaucoma optic nerve damage within 12 years [21].

Mechanical eyeball compression by the inferior rectus muscle is the main reason underlying increased IOP in patients with TAO with hypotropia [22]. Therefore, after inferior rectus muscle surgery, the additional force from the superior rectus muscle when upgazing can be eliminated. This allows easy eyeball rotation and the eyeball is not affected by thickened inferior rectus muscles. Mechanical compression of the muscles and superior rectus causes ocular hypertension. Moreover, we assessed IOP changes in 13 patients who underwent an inferior rectus recession. We found that that the pre- and postoperative mean IOP was 27.4 ± 4.6 mm Hg and 18.0 ± 2.7 mm Hg, respectively (difference: 9.4 ± 5.2 mm Hg). This further confirmed that mechanical compression by the thickened inferior rectus muscle is involved in IOP elevation during upgaze.

In conclusion, we observed normal IOP in patients with TAO with thickening of inferior rectus muscle while in the primary position. However, the IOP measured in clinical work is actually the upgaze transposition IOP in patients with TAO with hypotropia. Inferior rectus muscle thickening significantly increased the IOP when the eyeball was upgazed in patients with TAO compared to normal participants. This condition can be easily misdiagnosed as glaucoma, which suggests that IOP measurement in patients with TAO should be based on the primary position. For TAO patients with ocular hypertension, in addition to detailed inquiries regarding glaucoma-related history and related auxiliary examinations, the eye position should be given close attention to avoid misdiagnosis as glaucoma and the consequent administration of anti-glaucoma medication and surgery.

## Figures and Tables

**Table 1:** Demographic data.

	TAO group	Control group	Surgery group
n	36 (50 eyes)	33 (50 eyes)	13 (13 eyes)
Male	22 (27 eyes)	20 (28 eyes)	9 (9 eyes)
Female	14 (23 eyes)	13 (22 eyes)	4 (4 eyes)
Age (yr)	48.9 ± 9.8	43.3 ± 15.1	47 ± 9.4
Inferior rectus muscle (mm)	0.71 ± 0.13	0.36 ± 0.12	0.84 ± 0.10

**Table 2:** Comparison of IOP at different ocular positions between TAO group and control group.

	*N*	Primary position (mm hg)	Upgaze (mm hg)	Intraocular pressure difference (mm hg)
TAO	50	16.8 ± 2.1	25.7 ± 3.1	8.9 ± 2.7
Control	50	15.1 ± 1.9	17.6 ± 2.1	2.5 ± 1.4
*Z* value				−8.601
*P* Value				<0.0001

## Data Availability

The data used to support the findings of this study are available from the first author upon request.
